# Residents’ perspectives on a community health worker-delivered household air pollution prevention programme pilot in Eldoret, Kenya: a qualitative evaluation

**DOI:** 10.1080/16549716.2026.2641411

**Published:** 2026-03-09

**Authors:** Sepeedeh Saleh, Diana Menya, Maureene Ondayo, Noelle Sutton, Edna Sang, Sharon Cherono, Esilaba Anabwani, Nancy Chebichii, Serena Saligari, Daniel Pope, James Mwitari

**Affiliations:** a Department of Public Health, Policy & Systems, University of Liverpool, Liverpool, UK; b School of Public Health, Moi University, Eldoret, Kenya; c School of Environmental Studies, University of Eldoret, Eldoret, Kenya; d School of Public Health, Amref International University, Nairobi, Kenya; e Public Health & Health Systems, Kenya Medical Research Institute, Nairobi, Kenya

**Keywords:** Household air pollution (HAP), community health workers (CHWs), clean cooking solutions, clean energy, sustainable development goals (SDGs)

## Abstract

**Background:**

Household air pollution (HAP), from the use of polluting fuels for cooking, heating, and lighting, poses significant health and environmental risks, particularly in low-resource settings. The Community Household Air Pollution Prevention Programme (CHAP-PP) integrated a ‘household air pollution, health, and prevention’ module into Kenyan national Community Health Worker training, involving household-based education, awareness-raising, and discussions around air pollution, health, and clean energy.

**Objectives:**

This evaluation examined residents’ perspectives on the programme, considering impacts on energy use and HAP exposures in the context of wider experiences and providing recommendations for improvement.

**Methods:**

This qualitative descriptive study used semi-structured interviews and one focus group discussion with purposively selected household representatives, analysed using reflexive thematic analysis.

**Results:**

Residents welcomed the programme, reporting enhanced knowledge around HAP-related risks, harm mitigation practices (e.g. improved ventilation), and clean fuels. The latter enabled clean fuel adoption for some, but residual challenges remained. Using the COM-B (Capability-Opportunity-Motivation-Behaviour) model we considered how the programme influenced communities’ clean fuel uptake. Participants proposed solutions to financial barriers precluding clean fuel use including subsidies, value-added tax removal, and ‘pay-as-you-go’ schemes for liquefied petroleum gas, some of which have since been implemented.

**Conclusions:**

The CHAP-PP programme was felt to be successful, increasing HAP awareness and supporting harm mitigation and transitions to cleaner cooking. Findings have informed module rollout nationally and contributed to Kenya’s HAP prevention strategy. Ongoing efforts aim to improve affordability and scale clean cooking solutions, with future evaluations planned to assess long-term impacts on energy use and health outcomes.\

## PAPER CONTEXT

- **Main findings:** Household members reported improved awareness of household air pollution, clean fuels, and harm reduction following a novel community health worker-delivered household air pollution prevention programme in Kenya. Motivated to adopt clean fuels, participants identified residual challenges for community members and suggested potential solutions.
- **Added knowledge:** The study demonstrates how integrating household air pollution education in national community health worker programmes positively impacts household energy practices and knowledge, with evaluation findings guiding programme improvement.
- **Global health impact for policy and action:** The findings support scalable, community-based interventions as a strategy for improving community awareness of environmental determinants of health, such as air pollution and reducing the related health burdens in low-resource settings, and have already influenced Kenyan policy on clean fuel access.

## Background

Reliance on polluting fuels such as charcoal, crop residue, firewood, and kerosene for household energy represents a major global health and environmental priority. Globally, 3.8 billion people – almost half of the world’s population – depend on these fuels with exposure to household air pollution (HAP) from their combustion responsible for approximately 8.1 million deaths – including 700,000 deaths of children under 5, in 2021 alone [1]. Most people affected live in low- and middle-income countries, representing a significant source of global health inequality. HAP is causally associated with respiratory and cardiovascular diseases including chronic obstructive pulmonary disease (COPD), ischaemic heart disease, acute lower respiratory infection, lung cancer, and stroke [2,3]. HAP also affects pregnancies and infant growth and development, continuing transgenerational harm [3]. Women and children in rural regions are most directly exposed, and populations in sub-Saharan Africa bear a substantial proportion of the disease burden, with the least evidence of decline of all global regions [1,4,5].

In Kenya, 76% of the population relies on polluting fuels for cooking, compared with 30% globally, with those living in rural areas or informal settlements facing the greatest restrictions in access to clean cooking [6–8]. The Global Burden of Disease Study estimates around 26,000 lives lost annually in Kenya due to household air pollution exposure [9].

Clean fuels – as classified by the World Health Organization (WHO) – are those which produce fine particulate matter (PM2.5) and carbon monoxide (CO) levels aligned with WHO air quality guidelines [10,11]. This contrasts with ‘dirty’ or polluting fuels which pose a significant risk to health through emission of toxic substances. Clean fuels include Liquefied Petroleum Gas (LPG), kerosene, electricity, and solar energy. There is an urgent need to increase clean fuel use in Kenya and to mitigate risks associated with biomass use for those currently reliant on these fuels. In addition to air pollution exposure, open fires put women and children at risk of burns, and charcoal use is linked with asphyxiation, particularly in enclosed kitchens. Poisoning through accidental kerosene ingestion is a further significant risk [12–14].

Evidence indicates that improved knowledge and familiarity with clean fuels, alongside awareness of the harms of smoke from polluting fuels, are important in increasing the clean fuel uptake and supporting behaviour change to minimise health risks [15,16]. Community-based health interventions can be key to protecting community health by addressing the harms of polluting fuels. These programmes are delivered by trained community health workers (CHWs): members of local communities trained in health promotion and working at the interface between communities and health systems. Programme benefits arise primarily from promoting transitions to cleaner household energies such as LPG and solar energy [17]. Education around mitigating personal HAP exposure and related risks is also important, reducing harm in cases where use of polluting fuels is unavoidable. Mitigation measures may include improving ventilation or reducing burns risks.

Participants in a recent study in Rwanda highlighted the role of community-based education in facilitating a change of attitude towards household air pollution and encouraging safer cooking practices [18]. Recent examples of CHW involvement in African air pollution reduction include a combined water filter and air pollution intervention in Rwanda successfully employing CHWs to support the intervention, which also involved educational elements [19]. In Tanzania, locally trained Community Technology Workers were critical in overcoming barriers to LPG use relating to maintenance, education, and behaviour [20].

In Kenya, the community health system constitutes tier 1 of the national health system under Universal Health Coverage). Known as Community Health Promoters (CHPs), Kenyan CHWs are key in disseminating health messages to community members, each with a catchment area of one village. The 110, 000-strong CHP workforce covers the whole country, addressing health inequalities by reaching communities across diverse settings, including rural areas and informal settlements. This system provides an established network of trusted health workers embedded within communities, offering advantages over externally delivered programmes in terms of sustainability, credibility, and reach.

The Community Household Air Pollution Prevention Programme (CHAP-PP), developed by the CLEAN-Air(Africa) programme and partners (www.cleanairafrica.com) at the request of the Kenyan Ministry of Health, is an evidence-based training programme integrated into national CHP training as module 14 of the curriculum. The module includes a training manual, co-developed with CHPs and their communities, comprising four units on household energy use, health impacts of HAP, HAP prevention and safety, and health indicator surveillance. Corresponding ‘job aids’, including pictures, videos, maps, and a costing tool linked, are used by CHPs delivering the programme to facilitate household members’ understanding.

This paper presents findings from a qualitative evaluation of the CHAP-PP programme from household residents’ perspectives. The evaluation explored perceived impacts of the CHP-led sessions on communities’ understandings of the harms of polluting fuels, approaches to cleaner fuel adoption (primary prevention), and uptake of safety and harm-mitigation measures (secondary prevention). This inductive account of community perspectives also provides wider insights into residual barriers to clean fuel uptake and delivers contextually relevant recommendations from participants. Findings from this pilot implementation will guide work in the health and energy sectors to reduce reliance on biomass fuels and promote transitions to cleaner energy sources such as LPG, also informing national rollout of CHP training across all 47 of Kenya’s devolved counties.

## Methods

### Study setting and background

We report findings from the evaluation of a pilot implementation of the newly-developed Community Household Air Pollution Prevention (CHAP-PP) training programme in Kenya. CHAP-PP is the 14^th^ module developed by CLEAN-Air(Africa) for the Kenyan Ministry of Health to equip Community Health Workers to convey community-level health promotion messages to address the 26,000 lives HAP-attributable deaths annually in Kenya (almost 10% of all mortality). The training aims to deliver knowledge about household energy (safe use and combustion-related health impacts) and to start informed discussions with household members around ways of mitigating health risks (including HAP exposure). These ‘sensitisation sessions’ comprise both harm minimisation (reducing HAP exposure, for example through ventilation) and primary prevention by reducing emissions at source (adoption of clean fuels such as LPG, biogas, bioethanol, or electricity for cooking and lighting).

The pilot implementation took place with CHPs, formerly known as community health volunteers, from Langas sub-county: a peri-urban area largely comprised of informal settlements in Eldoret, Uasin Gishu County, Kenya. Women and children – mostly girls – are the primary cooks in most households, and charcoal and firewood are the most frequently used fuels for cooking, with a few households using both LPG and biomass fuels (‘fuel stacking’). Electricity is the main source of lighting, used by most households, but is replaced by candles and kerosene lamps during blackouts.

Community Health Promoters completed the three-day CHAP-PP (Module 14) training incorporating an instruction manual, job aids, and a ‘fieldwork’ day of observed community practice. The training was facilitated by Ministry of Health training facilitators and County Public Health personnel. The pilot implementation involved training 38 CHPs (27 females, 11 males) from 5 of the 6 sub-counties in Uasin Gishu County. Members of the Moi University CLEAN-Air(Africa) research team conducted a process evaluation of the training (knowledge acquisition and practice confidence). Following training, CHPs visited each of their 50–100 catchment households, delivering sensitisation sessions as part of their health promotion role.

### Study design and participant selection

This is a qualitative descriptive study, which employed individual semi-structured interviews and a focus group to assess perceptions of and outcomes relating to messaging and learning from the training using a case study approach [21]. A sample of nine households in Langas was purposively selected, and a key participant from each household invited to participate in semi-structured interviews and the final focus group discussion, with the same sample of participants taking part in both interviews and focus groups. The Langas area was selected partly in view of its proximity to the university, to avoid logistical challenges relating to travel, and also as it represented a typical peri-urban area, with residents primarily using biomass fuel, but with future potential to switch to clean fuels. Sampling of households and individuals for inclusion was supported by CHWs familiar with the structures of the local area and its households. A group of households and participants was identified and invited to take part in the study, with the selection designed to capture the usual diversity of household types and cooking practices in the community where the pilot implementation took place. This approach was used to provide insights into the typical range of community members’ responses to the programme [22]. Individual participant characteristics including age and education level were also varied in line with those commonly found in the local population. The predominance of women in our sample reflected those most likely to be involved in household cooking activities and interested in study participation.

Individual interviews were used to allow participants space to share their personal insights on the training and surrounding issues, including matters they may not have been comfortable openly discussing with other community members, such as those relating to household finances for example. The addition of a focus group discussion allowed for interactive discussions of key issues between participants, revealing common experiences and perceptions and allowing for deeper, shared exploration of certain themes. This relates to the concept of crystallisation [23], which describes the combining of multiple forms of data and perspectives to produce a nuanced understanding that resists singular interpretations.

### Data collection

Key features of the evaluation were explained to each participant and written informed consent was obtained before conducting the interviews and focus group discussion.

Household members were first interviewed in their homes and afterwards invited to a focus group discussion held in a local church, as a mutually agreed accessible and quiet location. Interviews and focus group discussions, based on collaboratively developed topic guides, were conducted by in Kiswahili by male and female members of the Kenyan research team trained in qualitative research methods, and audio recorded with participants’ consent. Verbatim transcription of the interviews and focus group discussion was carried out by research team members and the transcripts then reviewed by other members for accuracy. These were then loaded onto NVivo 12 for analysis.

### Data analysis

Reflexive thematic analysis [24] was used in view of its methodological flexibility and wide accessibility. The analytic approach was inductive, and data driven in nature, with no pre-existing framework in mind at the outset. This was in keeping with the wider interpretivist approach to the research, seeking to understand participants’ experiences and observations relating to household energy use and the programme’s impacts. Interview and focus group data were jointly analysed as they provided perspectives on the same questions, creating more comprehensive participant insights, with combined analysis adding to the credibility of the research findings [25].

Data were first reviewed by all members of the research team, who reflected on and discussed aspects of meaning and positionality. All members of the team carried out initial coding of transcripts, and codes were reviewed and discussed weekly within the team, allowing the gradual, collective, inductive development of a range of codes, then themes and sub-themes. The final results – in terms of themes presented in the paper – were also developed collaboratively by the group. This incorporation of different perspectives and reflexive discussion alongside the transparent development of themes all contributed to the dependability, and thus the confirmability of the process, further strengthening the final analysis [26,27].

## Results

Of the nine participants involved in individual interviews, seven were females and two males. Their ages ranged between 21 and 42 and a variety of educational backgrounds were represented (primary education to university-level education). Interviews lasted 21 minutes on average. The focus group discussion involved seven female and one male participants, as one of the initial participants was unable to attend the focus group discussion. The focus group discussion lasted 1 hour and 8 minutes.

Analysis of the focus group and interview data revealed interconnected themes relating to participants’ thoughts on the training programme and the issues it raised. The three overarching themes were as follows: appreciating how the programme built on existing knowledge about the harms of polluting fuels; residents learning about actions to reduce their HAP risks (subthemes here related to rethinking the accessibility of clean fuels leading some to switch to clean cooking and – for residents with a continued reliance on polluting fuels – advice around risk mitigation); and finally, participants’ approval of the programme leading them to recommend developments to extend its scope and impact, including programme-inspired suggestions for increased clean fuel accessibility. These themes collectively illustrate how the training programme influenced both individual decision-making and community-level advocacy for cleaner and safer household energy use.

### Acknowledging how the programme built on existing knowledge about the harms of polluting household fuels

Interview and focus group data often illustrated pre-existing knowledge about the health harms of smoke, as well as other risks associated with polluting fuels, amongst participants. Participants’ narratives demonstrated how the teaching sessions built on this knowledge, helping to bring the issues to the forefront in terms of understanding risks to families’ health. In terms of the latter, participants commonly cited carbon-monoxide-related asphyxiation risks from using charcoal in enclosed spaces and dirty walls from smoke-producing fuels. The following account from a resident reflects the experiences of several participants:

There is a day that we lit the *jiko* (charcoal-burning stove) when I did not know that charcoal is bad. As we were cooking, I saw my girl had fallen and begun to vomit. Now I took her out, I saw her vomiting, but she got well. They told me that perhaps we had locked ourselves in with the *jiko*. Truth be told, we had locked the door. That is when I knew that charcoal has harmful effects.

Interview, 42-year-old female household member

Whilst the information about smoke being harmful to health was rarely completely new, participants still described various benefits to this learning. Individuals had some knowledge in this area, to which the CHP programme added, deepening their understanding and bringing these issues to the fore. In talking about their new learning in this area, participants made links with incidents in their own lives and conversations they had had with others, demonstrating how the programme had developed their awareness. Further to this, it was clear that these links now motivated people to consider how they might take action to mitigate their smoke exposures.

The effects of adding new knowledge on the health impacts of cooking smoke were exemplified in the following quote from a female focus group participant:

Mostly I have come to know about pneumonia because I was born in the rural areas. Now there, almost the whole family complains about their chest and they use firewood. That it is when I came to relate (the two) and I said perhaps the cause is that smoke.

Interview, 37-year-old female household member

A participant with asthma became aware of the effects of her cooking on her symptoms following the conversations with CHPs, leading to decisions to transition to cleaner fuels:

I am asthmatic … at times when I use a *jiko* I get affected to the extent of taking medicine to calm me down. I am gradually shifting to cleaner sources of fuel.

Interview, 42-year-old female household member

### Gaining an understanding of actions to reduce household air pollution and related harms

Participants commonly described benefits they felt from the programme in terms of new understandings gained from CHPs about ways of reducing their HAP exposures and wider risks. This focused on knowledge around clean fuels such as LPG and around ways of mitigating the various harms from the use of polluting fuels.

#### Rethinking the accessibility of clean fuels, leading some residents to switch to clean fuels

When discussing potential transitions to clean fuels for household energy, CHPs initially introduced these fuels, particularly LPG which is the most accessible clean fuel in the community, with explanations of day-to-day aspects of fuel purchase and use. Participants often reported existing perceptions of LPG being too expensive, and unaffordable for them. In household visits, CHPs explained LPG pricing and supported household members in comparing the costs of using their current cooking methods and the likely costs of switching to LPG for household cooking. In this community, it is common to purchase solid fuels (e.g. charcoal) in small amounts (e.g. tins). When comparing the costs of these multiple purchases with bulk purchase of (for example) LPG, some residents described being pleasantly surprised at the affordability of LPG for cooking – particularly if income used for charcoal was saved for monthly LPG refills. For some, this was sufficient motivation to move to LPG cooking:

Before she taught me, I always assumed that they are expensive. I cannot meet that cost.

Some participants also brought together their new knowledge of the risks of solid fuels with these considerations of pricing, strengthening decisions to make a change to clean fuels:

When I came to compare that cost and I have come to learn of the side effects of charcoal, I just decide to move and shift to LPG.

Interview, 37-year-old female household member

Other residents explained that such saving was not possible in their current circumstances, often due to the ‘hand-to-mouth’ nature of their livelihoods. Such financial challenges were a widely stated barrier to LPG use by participants.

#### Recommending ways of improving access to clean fuels, in response to increased motivation to switch

Following the CHP sessions, participants described feeling motivated to change to LPG cooking but not being able to afford the outlay (cylinder deposit, regulator and hose, and burner), and cost of monthly refills. As one resident explained: ‘it is hard to save so that you can pay for LPG refills at the end of the month because we are dependent on everyday earnings and when you get like 40 shillings you buy charcoal and life goes on’. This participant, amongst others, recommended a scheme to allow the purchase of LPG fuel in small amounts:

the person who is selling a sack of charcoal and the one selling them in tins in small amounts, the one selling in tins has really helped us because we won’t have to buy a whole sack. So if there was something like that for gas refills it would be great.

Interview, 33-year-old female household member

Such ‘pay-as-you-go’ LPG schemes (using smart metres) are being trialled in Kenya and other sub-Saharan African countries, with positive outcomes (explored further in the Discussion) [28,29].

#### Planning additional ways of mitigating risks relating to polluting fuels

Interview and focus group data revealed various ways in which the CHP training sessions and community messaging helped household members to mitigate smoke exposure and reduce potential harm from using polluting fuels. These included messages around ventilation and smoke avoidance, such as opening windows and doors, taking charcoal stoves outside, and cooking away from children and vulnerable adults (e.g. sick and elderly) where possible.

One participant recognised, for example, that the process of lighting the fire led to particularly high levels of harmful smoke inhalation and described a plan to reduce this element of her personal exposure:

When I light the firewood, I leave so that it lights because I learnt that the more I sit there and blow, I continue to inhale that smoke, and the more I breathe in, it affects me.

Interview, 42-year-old female household member

Switching from firewood to charcoal, drying wood before use, and avoiding painted wood, which could release additional harmful fumes, were also mentioned, both as training content by CHPs as well as by household members who themselves suggested these mitigations.

These measures were additive to the learning around clean fuels, potentially reducing the negative health impacts of fuel stacking for those who used a mix of clean and polluting fuels. This was seen in the case of a household member who had switched from charcoal to LPG following the CHP health promotion session who, in response to a question around residual charcoal use, said: ‘I use it (charcoal) to cook *githeri* (mixture of beans and maize), and I ensure all the windows and door are open’ (Interview, 42-year-old male household member).

In addition to smoke mitigation, messages around safety were also well received. Participants described learning about preventing burns and accidents, which extended to paraffin ingestion by children from unattended bottles and fire risks relating to LPG use, as described by the following household member: ‘ … he (the CHP) told me when using LPG stove I should be careful with lighting it, I ensure there is no leakage and if I can smell the gas it means it is leaking and I should not light a matchstick’

### Recommending ways of further developing the programme and additional actions for promoting clean fuel adoption

In interviews and focused groups, participants praised the programme, suggesting various ways of furthering its reach and impact. Inspired by the programme’s message around clean cooking, participants also volunteered thoughts on improving clean fuel access for households, providing critical insights into the persisting barriers in this context.

#### Proposing programme additions and extensions

As stated, residents were enthusiastic about the CHP sensitisation sessions following CHAP-PP (Module 14) training and keen to share their learning with their family, friends, and neighbours. Participants suggested that the CHAP-PP programme be expanded, for sessions to be held in schools, churches, and other community areas, and for the programme to be formalised in various ways: ‘I suggest that this activity be incorporated into law and also be part of the government strategy and give(n) a pass on every entry level to the government.’ (Interview, 42-year-old male household member)

Whilst job aids used in the programme were widely appreciated, a few participants proposed that these resources be increased, for example, to include reading materials to be left at households:

It will be good if they can have several teaching aids whereby you will be given yours and they will be teaching while you have yours, you continue reading, and you will remain with it. You will be reading until it sticks, and you will help someone else.

Focus group, 31-year-old female household member

Some participants asked that CHPs be given t-shirts or caps to distinguish them in the community, and others suggested that they receive financial compensation for their work. Of note, a plan has since been announced by President Ruto for a programme to pay Kenyan CHPs a stipend for their health promotion work under Kenya’s Universal Health Coverage strategy [30].

#### Advocating for greater access to clean fuels for local communities

Going beyond their thoughts on existing programme delivery, household members commonly talked about how the clean cooking recommendations of the programme might be more widely reached, raising LPG accessibility, particularly from a financial perspective, as a key facet of this. Participants asked that LPG prices be reduced, for example, through subsidisation or reduction of VAT: ‘I plead to the government that many people are using LPG and they reduce the tax on LPG so that many people purchase LPG and shift from firewood or charcoal and go to LPG. Tax!’ (Focus group, 42-year-old male household member).

[At the time of the research VAT had been added to LPG as a post-COVID-19 cost recovery intervention. Research by CLEAN-Air(Africa) identified that this had led to discontinuation of clean cooking in Kenyan communities [7]. Subsequently (following a ministerial policy brief and cabinet memo) VAT was removed]

One participant, recommending the government subsidises LPG, cited savings in terms of reduced healthcare costs as an additional national benefit: ‘I would like to switch from the fuels I use to clean ones and I believe the government will benefit through reduced pressure in our healthcare systems if the community is enabled to make the switch’ (Interview, 33-year-old female household member).

Further to discussions around the difficulties of long-term saving for LPG, participants suggested schemes which allow the purchase of small amounts of LPG, similar to the system of tokens in place for electricity:

We are pleading with the government to pass the bill to introduce that LPG that you can use tokens and then we use. You know if it is cheap everyone will try to access it then the small amount that you have, you top up and cook with it then we will automatically shift from that smoke and move. Our cry is to the government.

Interview, 37-year-old female household member

One participant who had recently transitioned to LPG explained that this would help to ensure continued use of LPG, as well as new uptake by those currently using polluting fuels: ‘LPG refills are quite expensive. If there was a token LPG cylinder that you only pay up gas usage to the amount that one needs. Most of those already using LPG are complaining of high costs of refills’ (Interview, 39-year-old female household member).

## Discussion

This qualitative evaluation, from residents’ own perspectives, reported numerous felt benefits from the CHAP-PP initiative (delivered to CHPs as Module 14), which incorporated teaching and facilitated discussions around household energy use, safety, and health. Participants described how the sessions built on their knowledge and understanding of safe household energy use including the accessibility and benefits of clean fuels and reducing risks from polluting fuels through mitigation measures (harm minimisation). The programme stimulated a clear appetite for clean cooking amongst household members (typically adoption of LPG, given its accessibility within the community), which inspired participants to suggest ways of increasing uptake of clean cooking fuels within their communities.

**Figure 1.**
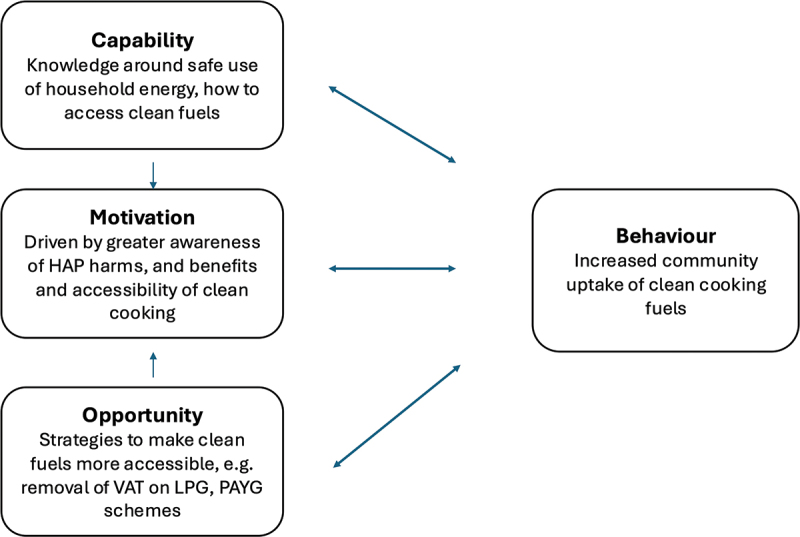
The COM-B model of behaviour change [31], with examples of factors specific to CHAP-PP initiative.

We now discuss key findings relating to programme impacts on clean fuel use with reference to elements of the COM-B model of behaviour change [31]. This framework defines three key categories – capability, opportunity, and motivation (COM) – required for behaviour (B) to occur. It provides a useful framework for understanding these components due to its clarity, and inclusion of relevant factors shaping individuals’ behaviours. Figure 1 reproduces the COM-B model, including key elements of the CHW programme’s contribution to each area.

HAP = Household Air Pollution; VAT = Value Added Tax; PAYG = ‘pay-as-you-go’.

We note, however, our use of the COM-B model only as an interpretive aid for elements of the discussion: it was not applied deductively in data analysis. This approach was used to enable a more open, data-driven analytic process, particularly important in this case in view of the contextually situated nature of the ‘behaviour’, rather than arguably simpler health-related behaviour changes such as medication adherence [32]. Beyond a linear analysis of barriers and facilitators to behaviour change, participants’ accounts touched on environmental, social, political, health, and economic aspects of their experience, which our discussion also assimilates.

The ‘Capability’ component of the COM-B model refers to ‘individual’s psychological and physical capacity’ to do the behaviour (including having the knowledge and skills). This was central to what participants felt the CHAP-PP initiative offered. Sessions provided community members with knowledge around the safe use of household energy and the harms of smoke from polluting fuels, and skills around the safe use of LPG, delivered by trusted community health representatives (CHPs). This is consistent with findings from the wider literature. For instance, a review of barriers and enablers of clean fuel adoption identified the importance of knowledge and skills around safe LPG use in overcoming fear of this technology [15], and a study in Sudan demonstrated how incorporating such training improved perceptions of, and therefore demand for, LPG for cooking [33]. Discussions with CHPs also helped household members to think through the possibilities of saving up their income, and to understand that – for some – this could make LPG a more accessible option than they had previously realised.

In terms of learning methods, Mezirow’s transformative learning theory [34] can help explain how the sessions supported this increase in ‘capability’ of the community members involved. The theory describes how teaching/learning experiences in adults link to individuals’ previous experiences (in this case, learning about cooking since childhood). This earlier learning (such as cooking experience) is then critically reviewed by the learner, integrating new information, through an active learning process. Here, teaching, discussions, and demonstrations CHPs sessions, alongside additional life experiences (for example where participants recalled adverse health experiences from smoke exposure or information previously gained from health staff), helped individuals to re-evaluate previous assumptions around smoke from cooking. Additional facets of the training sessions then supported the uptake of the required skills and knowledge (around, for example, adoption of clean fuels).

The role of CHPs was critical for the effectiveness of this health promotion messaging. The sensitisation sessions allowed CHPs to engage with community members in their household and involved the use of specially designed materials (job aids), demonstrations, as well as individual discussions. In a review of cookstove programmes, Lindgren [35] described the benefits of such community-integration, reaching beyond simple ‘education’ from an external source, and while this approach is relatively new, there is growing evidence of the successful use of CHPs in household interventions [36].

The ‘Motivation’ element of the model refers to reflective and automatic elements of motivation. This evaluation clearly demonstrated how the sensitisation sessions delivered by the trained CHPs increased individuals’ motivations to switch to adoption of cleaner fuels or to take actions to reduce exposures to minimise health harms. The automatic facet of this – which relates to emotional reactions, desires, impulses, and habits – was seen here in participants who, having learned about the benefits of clean cooking with LPG, were clearly inspired to try it. Researchers working in Cameroon found that positive perceptions about LPG amongst communities (in terms of safety, cooking speed and affordability, for example), were significantly associated with higher levels of exclusive LPG use [37], demonstrating the motivating value of nurturing positive perceptions of a new clean cooking technology. Individual discussions around household members’ individual situations, for example where parents described their children fainting from inhalation of air pollution (e.g. carbon monoxide), or others who pointed to dirty walls from smoke, created a reflective motivation to reduce emissions in the household (through clean fuels) or reduce levels/exposure through mitigation measures). This is linked with the educational elements discussed above, and creates an iterative cycle, increasing chances of behaviour change.

Finally, the ‘Opportunity’ facet is key to enabling change to occur, as skills, knowledge, and motivation alone cannot lead to change in the presence of insurmountable environmental barriers. Some analyses suggest that affordability is the most significant barrier to the uptake and continued use of clean fuels such as LPG for cooking [38]. This was in line with participants’ accounts in this study, which described how insecure livelihoods and irregular incomes made the prospect of saving for LPG refills difficult or even impossible (even without considering the outlay necessary for the initial cylinder/stove). Evidence from a trial of a ‘pay-as-you-go’ (PAYG) LPG solution in Kenya during the COVID-19 pandemic was consistent with these observations [29]. The study demonstrated how PAYG LPG allowed for continued use of LPG for clean cooking in households with income instability (induced by the pandemic through impacts on the informal economy), while those who did not have access to PAYG LPG (having to purchase the fuel in bulk), reduced use of the fuel (with the consequent re-introduction of health inequalities for those forced to revert to polluting fuels).

The CHAP-PP initiative and its evaluation provided community members with opportunities to advocate for better access to clean cooking with LPG, through reductions in charges and/or sale of smaller, more manageable quantities (e.g. PAYG LPG). There is clear evidence in the wider literature around the importance of so-called ‘structural interventions’ [39] – changes to economic, social, legal, political, or environmental factors shaping population health – in promoting health equity. Baum and Fisher describe how pressure from civil society can help build the necessary political will to create such changes [40,41]. As seen in this work, the integration of research with government policy and national health programmes provides strong opportunities for evidence-building and advocacy.

By addressing capability, motivation, and opportunity factors together, the CHAP-PP HAP, health and prevention initiative, works to maximise the possibility of changes to reduce and mitigate cooking-related exposure to household air pollution.

A key strength of the programme is in its delivery by trained community health workers to raise awareness of the health harms of household air pollution exposure and encourage consideration of cleaner fuels and mitigations. CHPs are from the communities they represent and experience the same health and social issues (including from reliance on polluting fuels). They are therefore trusted to impart evidence on the health benefits from reducing exposure to HAP and from primary prevention through clean household energy. The CHAP-PP initiative is one of the first in which community health workers are engaged in delivering HAP prevention sensitisation to household members as part of an overarching national CHW programme. Certain research trials have, however, employed CHWs in similar roles, as mentioned earlier in this paper [20,36]. Whilst slightly later in the intervention pathway, examples of CHWs supporting clean cooking and similar interventions demonstrate the strength of ‘community infrastructure’ approaches, which are increasingly being used in global health [42]. Looking forward, the community-health-based nature of this project, drawing on existing structures and staff, will enable follow-up over a longer period. Evaluations of programme implementation and outcomes, including use of quantitative indicators as introduced in the CHAP-PP initiative itself, will then inform iterative programme improvements and developments.

The project evaluation at this point has not integrated an in-depth consideration of gender, which constitutes a key limitation of the current qualitative evaluation. The household CHP sensitisation sessions and subsequent interviews and focus groups reported here involved a combination of male and female participants (more female than male, representing the usual distribution of cooking activity). The pilot implementation has not been able to incorporate an analysis of gendered aspects of the intervention: whilst primary cooks in Kenyan households are more often female, men may be equally or more involved in the household’s financial management. This is reflected in the household air pollution literature, and we agree with recommendations that comprehensive evaluations of this work include consideration of such factors as part of the analysis [35,43].

A final recommendation for the CHAP-PP initiative is for CHPs to re-visit households at a later point, ideally after the introduction of changes to improve the accessibility of clean cooking (e.g. with LPG or other solutions). These visits would allow CHPs to explore the uptake and continued use of cleaner cooking practices and clean fuels (for example whether they were being used exclusively or alongside other methods) and to understand residents’ lived experiences at this later stage, for example around any challenges which might be impeding their use. The literature on household cooking clearly indicates the variation of cooking needs according to different users, explaining how the consideration of these needs, and the possibility of adaptation for instance, to ensure that needs are met, are important determinants of continuing clean cooking [38,44,45]. This will constitute the next step of our work.

## Conclusions

This evaluation of the piloting of a ‘household air pollution, health and prevention’ module (Module 14), included within the CHP-delivered community health programme in Kenya, demonstrated extended knowledge amongst community members about the health harms of smoke exposure and mitigation (harm minimisation) and prevention (clean household energy) approaches. The success of the CHAP-PP initiative so far has led to Module 14 rollout being included in a recently published national HAP prevention strategy by the Ministry of Health in Kenya, supported by CLEAN-Air(Africa).

The initiative motivated residents to access clean fuels where possible, and participants in this evaluation have proposed contextualised solutions for widening access to these fuels in their communities. Recommendations around financial incentives and sale of PAYG LPG have recently been enacted in Kenya, and further strategies to scale clean cooking, including electric cooking, are being explored with communities. Future assessments of household energy use in the country will reveal the success of these actions in extending the scope of clean fuel access to the majority of Kenyans.

## Supplementary Material

CAA qual GHA_COREQ.xlsx

COREQ_Checklist_ss.pdf

Reflexivity statement_CHAP_PP_GHA.docx

## Data Availability

In view of the potentially sensitive or identifiable nature of the raw data, the data presented in this study are not freely available, but limited data may be available on request from the corresponding author.
