# Supplementary material for: Residents’ perspectives on a community health worker-delivered household air pollution prevention programme pilot in Eldoret, Kenya: a qualitative evaluation
Source: Glob Health Action. 2026 Mar 9;19(1):2641411. doi: 10.1080/16549716.2026.2641411 (PMC12973844; doi:10.1080/16549716.2026.2641411)
Supplement: Reflexivity statement_CHAP_PP_GHA.docx [file ZGHA_A_2641411_SM8806.docx]

**Reflexivity Statement**

**Evaluation of a Community Health Worker programme on household air pollution prevention: Transitioning to clean fuels and mitigating air pollution exposures in Eldoret, Kenya**

| **Study conceptualization** | 1. **How does this study address local research and policy priorities?**   This paper describes qualitative results of an evaluation of a household air pollution module integrated into community health worker training in Kenya, implemented by the Ministry of Health in Kenya as part of a national HAP Prevention Strategy. The work is led by national policy priorities and this evaluation will provide local perspectives from those directly involved with the initiative to inform future rollout of the programme. |
| --- | --- |
|  | 1. **How were local researchers involved in study design?**   Local researchers were integral to the inception, development, implementation, and outcomes of the initiative. In terms of the qualitative evaluation, Kenyan researchers led the focus groups and interviews, and a group of researchers of all levels were involved in formulating the paper, including data management and analysis and paper writing, as reflected in the authorship. |
| **Research management** | 1. **How has funding been used to support the local research team(s)?**   The research funding was used to provide salaries for local research staff involved, to allow research activities to go ahead, and to further development of local research capacity. |
| **Data acquisition and analysis** | 1. **How are research staff who conducted data collection acknowledged?**   The research staff working on these activities are named as authors of the paper. |
|  | 1. **How have members of the research partnership been provided with access to study data?**   Members of the research team have access to the data as appropriate, in accordance with their roles. Wider members of the partnership may be given access on request, subject to individual decisions, in view of the potentially sensitive nature of the qualitative data, to protect the confidentiality of participants. |
|  | 1. **How were data used to develop analytical skills within the partnership?**   Senior researchers set up and led a series of training sessions with research colleagues involving initial introductions to reflexive Thematic Analysis approaches followed by supported qualitative data management and collaborative analysis using the data to add capacity development aspects to the research. |
| **Data interpretation** | 1. **How have research partners collaborated in interpreting study data?**   As above, research analysis was collaborative, with reflexive discussion sessions throughout, to ensure the full range of perspectives were integrated into the analytic process. |
| **Drafting and revising for intellectual content** | 1. **How were research partners supported to develop writing skills?**   The drafting and editing of the manuscript was again collaborative. All authors had opportunities for contribution throughout the process of paper preparation. The team of more junior authors co-developed the initial draft, with support, and editorial input and advice was later sought from more senior partners. |
|  | 1. **How will research products be shared to address local needs?**   The insights from this research have already been integrated into policy-level change, and findings of the programme evaluation will directly inform future national implementation of the initiative. These results will also be made available to the wider global scientific community for discussion and to guide similar CHW-led work across the African region and further afield. |
| **Authorship** | 1. **How is the leadership, contribution and ownership of this work by LMIC researchers recognised within the authorship?**   The roles of researchers from Kenya (who make up a majority of the authorship of the paper) are recognised through authorship as stated, in keeping with the CreDiT taxonomy. |
|  | 1. **How have early career researchers across the partnership been included within the authorship team?**   Leadership of this paper was led by an early career researcher, who led training and development for a group of more junior researchers collaborating on the work (acknowledged through authorship) |
|  | 1. **How has gender balance been addressed within the authorship?**   The leading author and a majority of the overall team are female. Contributions are acknowledged in the authorsihp. |
| **Training** | 1. **How has the project contributed to training of LMIC researchers?**   Through the wider CLEAN-Air(Africa) research programme, staff (including those from LMICs) have ongoing training opportunities for supported research experience. The development and writing of this paper in particular was leveraged as a training opportunity for a group of staff – as reported in the response to Q6. The contributions of all staff involved has been recognised accordingly in the authorship. |
| **Infrastructure** | 1. **How has the project contributed to improvements in local infrastructure?**   The CLEAN-Air(Africa) research unit works to create improved access to clean energy for communities across Kenya, working with government, community groups, NGOs, and other relevant stakeholders, as well as large programmes of partnership research involving colleagues in Kenya and other LMICs. |
| **Governance** | 1. **What safeguarding procedures were used to protect local study participants and researchers?**   The study protocol was reviewed and approved by the relevant ethical committees. Only consenting participants participated, and participants were able to withdraw from the study, or elements of the study, at any point without pressure or adverse consequences. A named safeguarding lead was in place throughout the project, with clear avenues of contact for participants to report any concerns, and structures for appropriate referral of any such reports. |
